# Case of Simulated Neuralgia from a Common Pin, Which Had Been Swallowed, and in Eight Months after Emerged from the Surface of the Body

**Published:** 1851-10

**Authors:** H. C. Field


*Case of Simulated Neuralgia from a common Pin, which had been swallowed, and in eight months after emerged from the surface of the body.* By H. C. Field, M. D.—After some remarks on cases in which pins have been said to have been swallowed, or thrust into parts of the body, Dr. Field relates the following case:

Case. Mary M., a healthy woman, aged 30, in July, 1842, came to the Stillorgan Dispensary. She said her mouth had been full of pins: that she had accidentally swallowed one, which was choking her. Dr. Field immediately relieved her by using a probang. In the following October, he was requested to visit her as a dispensary patient; she then told him that she was in the greatest agony in her hip-joint, extending down the limb on the same side. He immediately bled her largely from the arm, had her cupped over the seat of pain, and gave her two grains of opium. The next day, she had some short sleep, but the pain was still very great. After exhausting every means he could think of, narcotising, mercurialising, blistering, and tonics, Dr. Field at length advised her to try a hospital. After submitting to various remedies in Steevens'

Hospital, without more than temporary relief, she was advised to go into the country for change of air, being greatly emaciated. She came again, greatly reduced in flesh, to the country, where Dr. Field saw her, but did nothing for her; and after remaining some time at home, suffering still from what appeared to be severe neuralgia, and being much reduced, she one day felt a sudden remission of the pain, and three days after it left her. From this time she rapidly recovered her health and strength, and was soon able to leave her bed. Shortly after, she found what she called a sore pimple on the inside of her thigh, which she poulticed and plastered, but could not cure; and having quite regained her strength, after an illness of *eight months*, she walked to the dispensary. On examining the pimple, Dr. Field found in its centre a sharp point; and after a little, he extracted, by its point, a large pin. Dr. Field thought that the simulation of idiopathic disease, for so long a time by a foreign body, rendered it extremely interesting, and established the *possibility* of a common pin, after being swallowed, emerging from the surface of the body.—*London Journal of Medicine.*

